# Association Between Fasting Hyperglycemia and New-Onset Atrial Fibrillation in Patients With Acute Myocardial Infarction and the Impact on Short- and Long-Term Prognosis

**DOI:** 10.3389/fcvm.2021.667527

**Published:** 2021-07-01

**Authors:** Mingxing Li, Yingying Gao, Kai Guo, Zidi Wu, Yi Lao, Jiewen Li, Xuansheng Huang, Li Feng, Jianting Dong, Yong Yuan

**Affiliations:** ^1^Department of Cardiology, Zhongshan People's Hospital, Zhongshan, China; ^2^School of Nursing and Health, Henan University, Kaifeng, China

**Keywords:** ST-segment elevation myocardial infarction, atrial fibrillation, fasting hyperglycemia, prognosis, mortality

## Abstract

**Background:** The relationship between fasting hyperglycemia (FHG) and new-onset atrial fibrillation (AF) in patients with acute myocardial infarction (AMI) is unclear, and whether their co-occurrence is associated with a worse in-hospital and long-term prognosis than FHG or AF alone is unknown.

**Objective:** To explore the correlation between FHG and new-onset AF in patients with AMI, and their impact on in-hospital and long-term all-cause mortality.

**Methods:** We performed a retrospective cohort study comprising 563 AMI patients. The patients were divided into the FHG group and the NFHG group. The incidence of new-onset AF during hospitalization was compared between the two groups and sub-groups under different Killip grades. Logistic regression was used to assess the association between FHG and new-onset AF. In-hospital mortality and long-term all-cause mortality were compared among patients with FHG, AF, and with both FHG and AF according to 10 years of follow-up information.

**Results:** New-onset AF occurred more frequently in the FHG group than in the NFHG group (21.6 vs. 9.2%, *p* < 0.001). This trend was observed for Killip grade I (16.6 vs. 6.5%, *p* = 0.002) and Grade II (17.1 vs. 6.9%, *p* = 0.005), but not for Killip grade III–IV (40 vs. 33.3%, *p* = 0.761). Logistic regression showed FHG independently correlated with new-onset AF (OR, 2.56; 95% CI, 1.53–4.30; *P* < 0.001), and 1 mmol/L increased in fasting glucose was associated with a 5% higher rate of new-onset AF, after adjustment for traditional AF risk factors. AMI patients complicated with both fasting hyperglycemia and AF showed the highest in-hospital mortality and long-term all-cause mortality during an average of 11.2 years of follow-up. Multivariate Cox regression showed FHG combined with AF independently correlated with long-term all-cause mortality after adjustment for other traditional risk factors (OR = 3.13, 95% CI 1.64–5.96, *p* = 0.001), compared with the group with neither FHG nor new-onset AF.

**Conclusion:** FHG was an independent risk factor for new-onset AF in patients with AMI. AMI patients complicated with both FHG and new-onset AF showed worse in-hospital and long-term all-cause mortality than with FHG or AF alone.

## Background

Hyperglycemia frequently complicates the clinical course of patients hospitalized with acute myocardial infarction (AMI) (1), and is associated with a worse prognosis regardless of diabetic status (2–4). Previous reports have shown that hyperglycemia causes oxidative stress, induces apoptosis, and activates coagulation, which exacerbates damage in the setting of ischemia (5–8). Atrial fibrillation (AF) is the most common supraventricular tachyarrhythmia in the general population (9, 10). It is also frequently observed after AMI, with a reported incidence of 5–23% (9, 11–13). AF is also a well-established predictor of poor short- and long-term prognosis in patients with AMI (9).

Although both hyperglycemia and AF are common in AMI patients, and extensive studies have indicated their value in predicting prognosis, little data are available concerning the correlation between hyperglycemia and new-onset AF. Whether hyperglycemia contributes to the prevalence of new-onset AF in this setting is unknown. Moreover, whether hyperglycemia combined with AF shows a worse prognosis than hyperglycemia or AF alone is unclear.

Therefore, we performed a retrospective cohort study with 10 years of follow-up data to explore the correlation between hyperglycemia and new-onset AF in patients with AMI, and their impact on in-hospital and long-term prognosis.

## Materials and Methods

### Study Population

We retrospectively enrolled patients who were admitted to our institute and diagnosed with AMI [including ST-segment elevation myocardial infarction (STEMI), and non-ST-segment elevation myocardial infarction (NSTEMI)] from 1 January 2007 to 1 January 2009. The ethics committee on clinical scientific research and laboratory animal of Zhongshan People's Hospital approved the study (review number: K2019-057). The exclusion criteria were patients (1) with previous known AF or atrial flutter, (2) with unavailable hyperglycemia data, (3) complicated with pulmonary failure defined as a PO_2_ level <60 mmHg or a PCO_2_ level more than 50 mmHg according to blood gas analysis, (4) complicated with liver damage defined as an ALT level 3 times higher than the normal upper limit, (5) complicated with severe renal failure defined as a serum creatinine (CR) level >265 μmol/L (6) complicated with pancreatic system diseases or heart valve disease (moderate to severe valve regurgitation or stenosis), (7) complicated with hyperthyroidism, and (8) complicated with carcinoma.

### Methods

#### Baseline Data Collection

Data on age, sex, previous history of diabetes, hypertension, cerebral stroke, and smoking habits were collected according to inpatient records. Other data, such as the peak value of creatine kinase MB isoenzyme (CK-MB), CR, fasting glucose (FG) level, glycosylated hemoglobin level, left atrial diameter, and left ventricular ejection fraction (LVEF) according to echocardiography, Killip grades, medication treatment during hospitalization, whether the percutaneous coronary intervention (PCI) was performed and new-onset AF complications, were also collected.

#### Correlation Between Hyperglycemia and New-Onset AF

All the enrolled patients were divided into a fasting hyperglycemia group (FHG group, FG ≥ 7 mmol/L), and a no fasting hyperglycemia group (NFHG group, FG <7 mmol/L). The incidence of new-onset AF during hospitalization was compared between the two groups as well as sub-groups under different Killip grades.

#### In-Hospital Mortality

The patients were divided into four groups according to whether they had AF, hyperglycemia or both: FHG–AF– (have neither hyperglycemia nor AF), FHG–AF+ (have AF only), FHG+AF– (have hyperglycemia only), and FHG+AF+ (have both hyperglycemia and AF). In-hospital mortality among the four groups was compared.

#### Ten Years of Follow-Up

We choose all-cause mortality as the endpoint of the study. All patients who survived and were discharged were followed up until December 31, 2019. We collected follow-up information either by outpatient medical records or through routine telephone follow-up records every year. Death events were recorded during this period. We compared the influence of FHG and new-onset AF, and their co-occurrence on all-cause mortality.

### Definition of Exposure and Outcomes

#### Definition of New-Onset AF

AF was diagnosed as the absence of P waves, coarse or fine fibrillatory waves, and irregular RR intervals. New-onset AF was defined as electrocardiographic evidence of AF during hospitalization, and those patients were absent of persistent or paroxysmal AF or atrial flutter previously.

#### Definition of AMI

AMI was defined as patients with typical angina pectoris and ST-elevation at the J-point with the cutoff point of C 0.1 mV in at least two contiguous leads of the electrocardiogram (ECG) in standard lead I through AVF. An ST-elevation of 0.2 mV in V1 through V6 was defined as STEMI, and patients with increased cardiac biomarkers (mainly creatinine phosphokinase, CPK) were defined as NSTEMI.

#### Definition of Hyperglycemia

In our study, we chose the FG concentration as the evaluation index, and samples were measured upon hospital admission with at least 8 h of overnight fasting. A cutoff value of 7 mmol/L (126 mg/dl) was used to define fasting hyperglycemia.

### Statistical Analysis

Statistical analysis was conducted with Statistical Package for the Social Sciences (SPSS) 17.0 software (SPSS Inc., Chicago, IL, United States). Numerical variables are represented as the mean ± standard deviation or median, and categorical variables as percentages or rates. To test differences between the groups, the Student's *t*-test was used for numerical variables with a Gaussian distribution, and the Mann–Whitney *U*-test was employed if there was a non-Gaussian distribution. Categorical variables were analyzed with the chi-squared and Fisher's exact tests. Logistic regressions were used to assess the relationship between FG and new-onset AF. We primarily modeled FG as a continuous variable, and then categorized FG into hyperglycemia group (FG ≥ 7.0 mmol/L) and normal group (FG <7.0). The initial model was adjusted for age and gender. A second model was additionally adjusted for smoking, hypertension, previous MI, previous stroke, and creatine. Kaplan-Meier analysis was used to compare long-term all-cause mortality under different FHG and new-onset AF status. Cox regression analysis was performed to identify risk factors for all-cause mortality. A *p* < 0.05 was regarded as statistically significant.

## Results

### Enrollment and Baseline Characteristics of the Study Population

**Figure 1:**
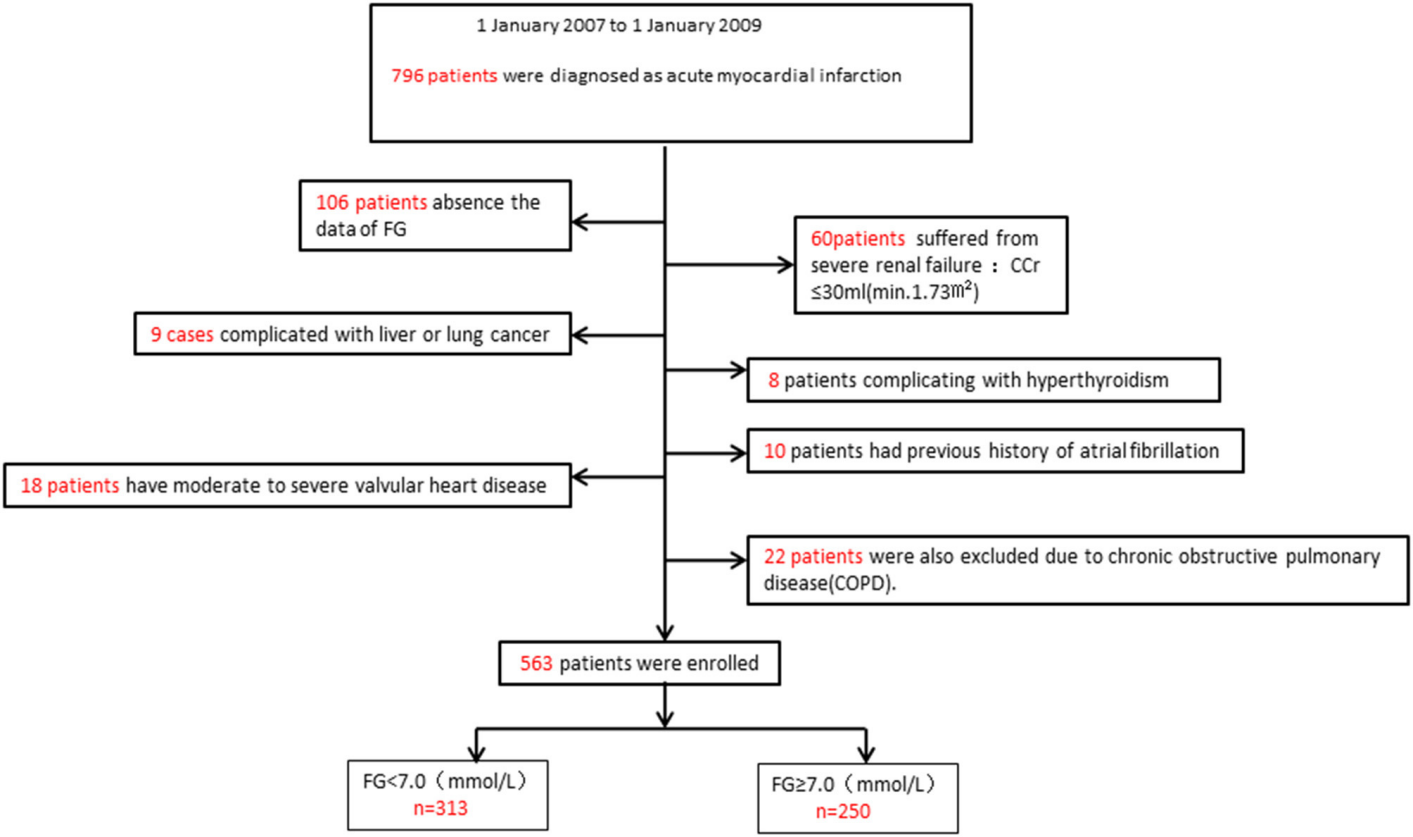
Flow chart of participants. FG, fasting glucose; Ccr, creatinine clearance rate.

From 1 January 2007 to 1 January 2009, a total of 796 patients diagnosed with AMI were admitted to our institute. Five hundred sixty-three patients were finally enrolled in this study. Two hundred fifty patients were assigned to the FHG group (FG ≥ 7.0 mmol/L), and 313 patients were assigned to the NFHG group (FG <7.0 mmol/L). The patient flowchart is shown in Figure 1.

Of the 563 patients enrolled, 448 (79.6%) were male, and the average age was 62.59 ± 13.20 years. AMI patients were treated according to guidelines recommendation. Eighty-three patients (14.7%) were complicated with new-onset AF. Fifty-two cases of which were paroxysmal AF while the other 31 were persistent AF. All 52 cases with paroxysmal AF successfully recovered sinus rhythm spontaneously. Twenty-five of the other 31 persistent AF were also converted to sinus rhythm either by direct-current, or by pharmacological cardioversion. Individualized antithrombotic therapy was also given to patients according to the guidelines recommendation and clinical evaluation. Participants in the FHG group were more likely to be female, non-smoker, lower LVEF, higher Killip classification, and previous DM (Table 1).

**Table 1:** Baseline characteristics of patients in NFHG group vs. FHG group.

	**NFHG group (*n* = 313)**	**FHG group (*n* = 250)**	***P*-value**
**General Characteristics**			
Age, years	62.04 ± 13.15	63.29 ± 13.25	0.306
Gender, male,%	265 (84)	183 (73)	0.003^*^
**Previous History**			
Myocardial infarction, *n*, %	3 (0.9)	7 (2.8)	0.89
Angina pectoris, *n*, %	5 (1.6)	8 (3.2)	0.26
Cerebral stroke, *n*, %	19 (3.1)	11 (4.4)	0.45
Diabetes mellitus, *n*, %	26 (8.3)	45 (18)	0.001^*^
Smokers, *n*, %	178 (56.9)	108 (43.2)	0.003^*^
Hypertension, *n*, %	137 (43.8)	114 (45.6)	0.608
**Biochemistry and Image Results**			
Peak value of CK-MB, U/L	129.9 ± 79.4	146.0 ± 39.8	<0.001^*^
Killip grade I, *n*, %	217 (69.3)	141 (56.4)	<0.001^*^
Left ventricular ejection fraction	0.52 ± 0.01	0.50 ± 0.01	0.001^*^
Left atrial diameter, mm	35.5 ± 6.6	34.8 ± 8.6	0.201
Serum potassium, mmol/L	3.83 ± 0.38	3.80 ± 0.43	0.075
**Medication During Hospitalization**			
Aspirin, *n*, %	295 (94.2)	225 (90)	0.078
Clopidogrel, *n*, %	270 (86.2)	208 (83.2)	0.334
Statins, *n*, %	300 (95.8)	230 (92)	0.070
Low molecular weight heparin, *n*, %	248 (79.2.3)	195 (78)	0.756
Warfarin, *n*, %	38 (12.1)	50 (20)	0.014^*^
β blocker, *n*, %	137 (43.8)	114 (45.6)	0.391
ACEI, *n*, %	304 (97.1)	219 (87.6)	<0.001^*^
GPIIb/IIIa, *n* %	54 (17.3)	59 (23.6)	0.057
Diuretics, *n*, %	177 (56.5)	199 (79.6)	<0.001^*^
**Reperfusion Therapy**			
Acute anterior myocardial infarction, *n*, %	110 (35.1)	80 (32)	0.531
Patients which PCI were performed, *n*, %	247 (68)	178 (70)	0.076
Triple vessels or left main leision	117 (37.4)	84 (33.6)	0.427
Rate of new onset atrial fibrillation	29 (9.2)	54 (21.6)	<0.001^*^

*NFHG, no fasting hyperglycemia; FHG, fasting hyperglycemia; ACEI, renin angiotensin converting enzyme inhibitor; PCI, percutaneous coronary intervention*;

* *P < 0.05*.

### Correlation Between Fasting Hyperglycemia and New-Onset AF in Patients With AMI

New-onset AF occurred more often in the FHG group than in the NFHG group (21.6 vs. 9.2%, *p* < 0.001), Table 1. The FHG group showed worse pump function than the NFHG group. We performed subgroup analysis according to different Killip classifications to further clarify the relationship between hyperglycemia and AF. In subgroup Killip grade I, the FHG group had a higher rate of new-onset AF than the NFH group (16.6 vs. 6.5%, *p* = 0.002), and a similar trend was observed in subgroup Killip grade II (17.1 vs. 6.9%, *p* = 0.005), but not for Killip grade III–IV (40 vs. 33.3%, *p* = 0.761). Table 2 shows the comparison of baseline variables and incidence of AF among groups.

**Table 2:** Sub-group analysis.

	**Killip grade I**		**Killip grade II**		**Killip grade III–IV**	
	**NFHG *n* = 216**	**FHG *n* = 140**	**NFHG *n* = 82**	**FHG *n* = 70**	**NFHG *n* = 15**	**FHG *n* = 40**
Gender, male, %	186 (86.1)	101 (72.1)^*^	65 (79.3)	53 (75.7)	14 (93.3)	29 (72.5)
Age, year, *$\bar{x}$* ±*s*	61.8 ± 12.8	63.2 ± 13.2	64.2 ± 12.2	65.0 ± 12	65.7 ± 4.1	64.0 ± 2.4
Left ejection fraction *$\bar{x}$* ±*s*	0.54 ± 0.11	0.51 ± 0.13	0.50 ± 0.11	0.46 ± 0.12	0.46 ± 0.11	0.45 ± 0.13
Serum potassium *$\bar{x}$* ±*s*	3.82 ± 0.40	3.80 ± 0.42	3.82 ± 0.36	3.82 ± 0.46	3.79 ± 0.46	3.78 ± 0.42
Left atrial diameter *$\bar{x}$* ±*s*	34.8 ± 5.8	34.1 ± 8.3	37.1 ± 8.1	36.2 ± 8.0	34.8 ± 5.8	34.1 ± 8.3
Diabetes, *n* %	16 (7.4)	28 (20.0)	8 (9.8)	8 (11.4)	2 (13.3)	9 (22.5)
Hypertension, *n*%	94 (43.5)	64 (45.7)	38 (46.3)	33 (47.1)	5 (33.3)	17 (42.5)
Rate of new onset fibrillation, *n*, %	14 (6.5)	23 (16.4)^*^	10 (12.2)	15 (21.4)^§^	5 (33.3)	16 (40)

*Baseline characteristic and new onset AF comparation between NFHG group and FHG group under different killip level*.

* *p < 0.01*;

§ *p < 0.001*.

We performed a multivariate logistic regression analysis in model 1 to model 3 by subsequently adjusting confounding risk factors to explore whether FHG was independently correlated with new-onset AF. In the model that measured FG as a continuous variable, an increase of 1 mmol/L in FG was associated with a 5% higher rate of new-onset AF [(odds ratio, 1.05; 95% CI, 1.00–1.10); *P* = 0.044], after adjusting for all co-variables (Table 3). Results were similar when we categorized individuals into the FHG and NFHG groups in both unadjusted and adjusted models (*P* < 0.001). In the final model, the odds ratio for the FHG group was 2.56 (95% CI, 1.53–4.30; *p* < 0.001), taking the NFHG group as reference (Table 3).

**Table 3:** Association between fasting blood glucose and new onset atrial fibrillation.

**FBG**	**Incident rate**	**Model 1**		**Model 2**		**Model 3**	
		**OR (95% CI)**	***P*-value**	**OR (95% CI)**	***P*-value**	**OR (95% CI)**	***P*-value**
Continuous variables, per mmol/L	83/563	1.07 (1.02, 1.12)	0.006	1.06 (1.01, 1.11)	0.016	1.05 (1.00, 1.10)	0.044
≥7 mmol/L	55/250	2.88 (1.75, 4.74)	<0.001	2.79 (1.68, 4.63)	<0.001	2.56 (1.53, 4.30)	<0.001
<7 mmol/L	28/313	Reference		Reference		Reference	

*Model 1, adjusted for age, gender; Model 2, further adjusted for smoking, hypertension, previous MI, previous stroke, creatine; Model 3, further adjusted for previous DM, ejection fraction*.

### Incidence of New-Onset AF Among Stress Hyperglycemia, Newly Diagnosed DM, and Previously Diagnosed DM

**Figure 2:**
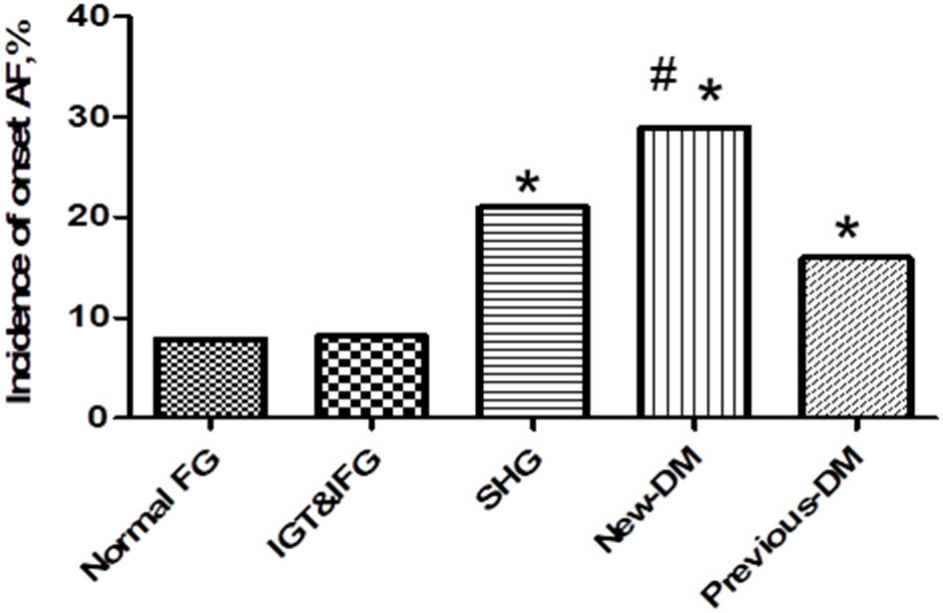
Incidence of new-onset atrial fibrillation among stress hyperglycemia (SHG group), newly diagnosed diabetes (New-DM group), and previous diabetes (previous-DM group). Previous-DM patients, newly diagnosed DM patients and SHG patients had a higher rate of new-onset fibrillation compared with patients with normal FG group (16 vs. 29 vs. 21 vs. 7.8%,*P < 0.05), while there is no statistic difference among the three groups (16 vs. 29 vs. 21%, ^#^*p* = 0.152).

According to the levels of fasting blood glucose, 2 h postprandial blood glucose (determined by the oral glucose tolerance test) and glycosylated hemoglobin (HbA1c), patients were divided into the following groups: previously diagnosed with diabetes mellitus (previous DM), newly diagnosed with diabetes mellitus (N-DM) and stress hyperglycemia (SHG). The previous DM, N-DM, and SHG patients had a higher rate of new-onset AF than patients in the normal FG group, while there was no significant difference among the previous DM, N-DM, and SHG groups (Figure 2).

### In-Hospital and Long-Term Mortality

**Figure 3:**
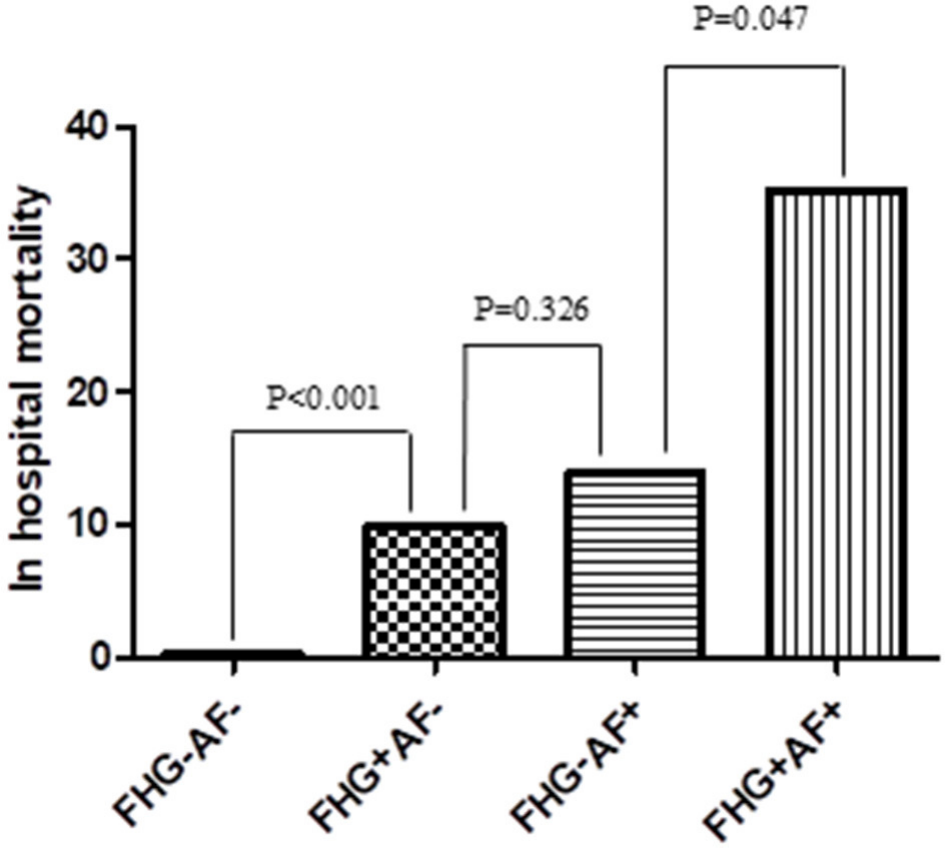
Comparison of in hospital mortality among groups. FHG+AF– group showed higher rate of in hospital mortality compared with FHG–AF– group (9.7 vs. 0.3%, *P* < 0.001). In hospital mortality was higher in FHG+AF+ group compared with FHG–AF+ group (35.2 vs. 13.8%, *P* = 0.047), while there was no difference between FHG+AF– and FHG–AF+ group (9.7 vs. 13.8%, *P* = 0.326). FHG–AF–, patients with neither fasting hyperglycemia nor new-onset atrial fibrillation; FHG+AF– patients with fasting hyperglycemia but without new-onset atrial fibrillation; FG–AF+, patients with new-onset atrial fibrillation but without fasting hyperglycemia; FG+AF+, patients with both fasting hyperglycemia and new-onset atrial fibrillation.

Either hyperglycemia or new-onset AF contributed to the increased in-hospital mortality in patients with myocardial infarction. Compared with the other patients, those complicated with both FHG, and new-onset AF exhibited the highest in-hospital mortality (Figure 3). Multivariate logistic regression analysis showed that both new-onset AF and FHG were independent risk factors for in-hospital mortality after adjusting other traditional risk factors (Table 4).

**Table 4:** Logistic regression analysis for risk factors attributing to in-hospital mortality in the study.

	**Univariate analysis**		**Multivariate analysis**	
**Variable**	**OR (95% CI)**	***P*-value**	**OR (95% CI)**	***P-*value**
Age, years	1.019 (0.994–1.045)	0.132		
Gender, male	0.633 (0.314–1.276)	0.201		
History of HBP	2.362 (1.228–4.547)	0.01		
History of DM	1.658 (0.736–3.735)	0.222		
Killip grade II–IV	3.161 (1.660–6.019)	<0.001^*^	1.446 (0.647–3.232)	0.369
Peak value of CK-MB	1.000 (0.999–1.001)	0.938		
With left main or tripple vessels lesions	0.520 (0.251–1.079)	0.079		
Left ejection fraction	0.007 (0.001–1.067)	<0.001^*^	0.031 (0.002–0.533)	0.017^*^
Left atrium diameter	0.989 (0.948–1.031)	0.594		
Complicated with FHG	11.130 (4.130–28.741)	<0.001^*^	8.134 (2.664–24.841)	<0.001^*^
With new onset AF	6.580 (3.400–12.736)	<0.001^*^	6.612 (2.878–15.191)	<0.001^*^
PCI performed	0.348 (0.184–0.658)	0.001^*^	0.583 (0.257–1.322)	0.196
Use of ACEI/ARB	0.097 (0.045–0.208)	<0.001^*^	0.342 (0.114–1.024)	0.055
Use of β-blocker	0.213 (0.108–0.420)	<0.001	0.226 (0.081–0.628)	0.044^*^
Use of diuretics	0.890 (0.502–1.581)	0.692		

*DM, diabetic mellitus; HBP, hypertensive blood pressure; CK-MB, creatine kinase-MB isoenzyme; FHG, fasting hyperglycemia; AF, atrial fibrillation; PCI, Percutaneous coronary intervention; ACEI, angiotensin converting enzyme inhibitor; ARB, angiotensin receptor blocker*;

* *P < 0.05*.

**Figure 4:**
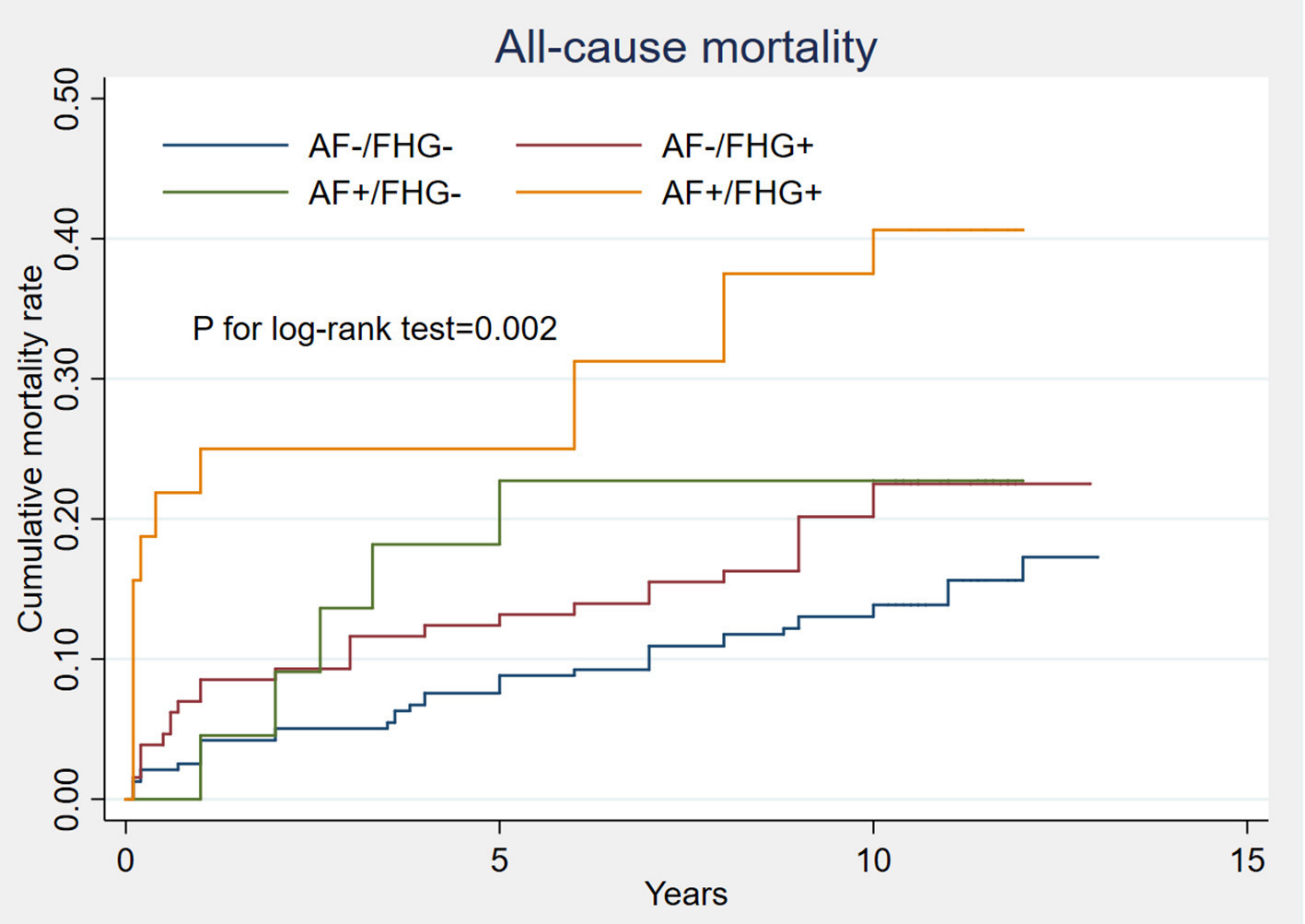
Comparison of long term all-cause mortality under different FHG and new-onset AF status. Kaplan-Meier analysis showed that patients complicated with either FHG (AF–/FHG+) or AF (AF+/FHG–) had obviously higher cumulative rates of all-cause mortality than those with neither AF nor FHG (AF–/FHG–). Patients complicated with both FHG and AF (AF+/FHG+) showed the worst long-term all-cause mortality, *P* for log-rank test was 0.002.

A total of 520 patients were discharged except for 43 deaths during hospitalization. Forty-two other cases were lost with follow-up information and 94 deaths were recorded during an average of 11.2 ± 0.68 years of follow-up. Kaplan-Meier analysis showed that patients complicated with either FHG or AF showed a higher cumulative rates of all-cause mortality than those with neither AF nor FHG. Patients complicated with both FHG and AF showed the highest long-term all-cause mortality (*P* for log-rank test = 0.002, Figure 4). Multivariate Cox regression showed that AMI patients with both FHG and AF (FHG+AF+) were independently correlated with long term all-cause mortality after adjusting other confounding risk factors (odds ratio, 3.13; 95% CI 1.64–5.96, *P* = 0.001, Table 5).

**Table 5:** Association between AF/FHG status and all-cause mortality.

**AF/FH status**	**Incident rate**	**Model 1**		**Model 2**		**Model 3**	
		**HR (95% CI)**	***P*-value**	**HR (95% CI)**	***P*-value**	**HR (95% CI)**	***P-*value**
AF–/FHG–	40/266	Reference		Reference		Reference	
AF–/FHG+	33/148	1.47 (0.90, 2.42)	0.123	1.46 (0.88, 2.40)	0.142	1.41 (0.85, 2.35)	0.184
AF+/FHG–	6/27	1.51 (0.59, 3.83)	0.390	1.61 (0.63, 4.12)	0.317	1.44 (0.56, 3.70)	0.453
AF+/FHG+	15/37	3.76 (1.99, 7.09)	<0.001	3.45 (1.82, 6.54)	<0.001	3.13 (1.64, 5.96)	0.001

*AF, atrial fibrillation; FHG, fasting hyperglycemia*.

*Model 1, adjusted for age, gender; Model 2, further adjusted for smoking, hypertension, previous myocardial infraction, previous stroke; Model 3, further adjusted for previous DM, ejection fraction*.

## Discussion

To the best of our knowledge, this is the first study to systematically explore the relationship between FHG and new-onset AF, and their combined impact on prognosis in patients with AMI with a long-term follow-up. The main findings of the article are as follows: (i) fasting hyperglycemia was independently associated with new-onset AF after adjusted other traditional risk factors in patients with AMI. (ii) AMI patients complicated with both FHG and new-onset AF showed worse in-hospital mortality and long-term all-cause mortality than FHG or AF alone.

AF is the most common super-ventricular tachyarrhythmia in the general population (9, 10). It is also frequently complicating the clinical course after an acute myocardial infarction with a reported incidence of 5–23% (9, 11–13). In this study, we found that 81 of 549 (14.8%) AMI patients complicated with new-onset AF. Our result was consistent with previous reports (9, 11–13). Several clinical variables such as age, congestive heart failure, kidney disease, hypertension, diabetes, and pulmonary disease have been reported as risk factors for new-onset AF (12, 14–18). However, whether FHG was an independent risk factor for new-onset AF in AMI patients was not established. Several large sample size prospective epidemiologic studies based on nationwide or community databases have showed that, in the general population, FG was independently correlated with AF (19–21). However, whether FG remained an independent risk factor for new-onset AF in AMI has not been reported. In this study, we found new-onset AF occurred more often in the FHG group than in the NFHG group, and this association was consistent in several subgroup analyses under different Killip grades. To further clarify the relationship, we subsequently adjusted confounding risk factors such as age, gender, smoking, hypertension, previous MI, previous stroke, creatine, previous DM and ejection fraction, and found that FG remained an independent risk factor for AF. We then categorized individuals into FHG group and normal group, the results were similar.

AMI patients complicated with hyperglycemia contained three clinical status: prior or new diagnosed diabetes, prior or newly diagnosed pre-diabetes and stress hyperglycemia (2). In our study, we compared the rate of new-onset AF among (SHG), newly diagnosed diabetes (new-DM), previously diagnosed diabetes (previous-DM), prior or newly diagnosed pre-diabetes group. We found that DM, N-DM, and SHG patients had a higher rate of new-onset AF than the normal FG-group or pre-diabetes group, while there was no significant difference among the DM, N-DM, and SHG groups. After adjustment for previous diabetes and other confounding risk factors, FHG independently correlated with new-onset AF (Table 3). Our results may be valuable in reminding clinicians to pay more attention to the short-term fluctuation in fasting blood glucose after AMI.

The exact mechanism by which hyperglycemia is closely related to an increased incidence of new-onset AF in AMI is still unclear, and several reasons may explain this. First, in AMI, the strong excitation of the sympathetic adrenal medulla releases a large amount of catecholamines, glucagon hormones and growth hormones, which can reduce glycogen synthesis and glucose utilization in myocardial cells (22). In addition, hyperglycemia leads to platelet aggregation, fibrinolysis inhibition, and collateral circulation disturbance, which increases the imbalance of myocardial energy supply and demand (6, 23, 24). This affects the polarization of cardiac myocytes and is associated with various arrhythmias, including AF (25). Second, the hyperosmotic state of blood caused by hyperglycemia has an important impact on the electrophysiological activity of atrial myocytes in circumferential pulmonary veins, which may also be one of the important reasons for the increased incidence of AF (26). Evidence from animal experiment showed that hyperglycemia can lead to spatial heterogeneity (nerve remodeling) of autonomic innervation of atrial myocytes, which was also a potential mechanism of increased incidence of AF (27). Finally, part of AMI patients complicated with FHG is due to DM and DM is associated with an increased risk of subsequent AF (28, 29). Other FHG patients may be with a higher incidence of metabolic syndrome and non-alcoholic fatty liver disease (NAFLD), which is also associated with the risk of AF (30).

There was sufficient clinical evidence to show that either hyperglycemia or new-onset AF were correlated with poor prognosis in patients with AMI (2–5, 9–11); pre-diabetes was also reported as an important risk factor for all-cause mortality (31). However, there are no data concerning whether hyperglycemia combined with new-onset AF adds more weight to a poor prognosis in AMI patients. In this study, we found AMI patients with either FHG or AF showed higher all-cause mortality, and patients with both FHG and AF increased three times risk of all-cause mortality after adjusting other confounding risk factors. This may guide our clinical practice: if FHG can be controlled to a certain level, it may decrease the incidence of new-onset AF and long-term all-cause mortality. Larger sample sizes and prospective clinical trials are needed to clarify this problem in the future.

## Limitations

There are several limitations of this study. First, it is a single center retrospective cohort study and it has the defect of missing follow-up. Further prospective studies with larger sample sizes should be conducted to explore the relationship between FG and new-onset AF in patients with AMI. Second, as an observational study, we could not exclude residual confounders, despite being adjusted for potential covariates as much as possible. Finally, we record new-onset AF only by ECG, which may underestimate the incidence of AF in the real world. However, the incidence of new-onset AF in our study is similar to other previous studies (9, 11–13).

## Conclusions

FHG was an independent risk factor for new-onset AF in patients with AMI. AMI patients complicated with FHG and new-onset AF showed worse in-hospital and long-term all-cause mortality than those with FHG or AF alone.

## Data Availability Statement

The original contributions presented in the study are included in the article/supplementary material, further inquiries can be directed to the corresponding author.

## Ethics Statement

The studies involving human participants were reviewed and approved by Ethics committee on Clinical scientific research and laboratory animal of Zhongshan People's Hospital. Written informed consent for participation was not required for this study in accordance with the national legislation and the institutional requirements.

## Author Contributions

ML wrote and edited the manuscript. YG, KG, ZW, YL, and JL collected the research data for the article. LF, JD, XH, and YY reviewed the manuscript and approved the final manuscript. All authors contributed to the article and approved the submitted version.

## Conflict of Interest

The authors declare that the research was conducted in the absence of any commercial or financial relationships that could be construed as a potential conflict of interest.

## Abbreviations

AMI: acute myocardial infarction
AF: atrial fibrillation
FHG: fasting hyperglycemia
NFHG: non- fasting hyperglycemia
FG: fasting glucose
DM: diabetes mellitus.
